# Automated Forensic Animal Family Identification by Nested PCR and Melt Curve Analysis on an Off-the-Shelf Thermocycler Augmented with a Centrifugal Microfluidic Disk Segment

**DOI:** 10.1371/journal.pone.0131845

**Published:** 2015-07-06

**Authors:** Mark Keller, Jana Naue, Roland Zengerle, Felix von Stetten, Ulrike Schmidt

**Affiliations:** 1 Laboratory for MEMS Applications, IMTEK–Department of Microsystems Engineering, University of Freiburg, Freiburg, Germany; 2 Hahn-Schickard, Freiburg, Germany; 3 Institute of Legal Medicine, Freiburg University Medical Center, Freiburg, Germany; 4 Faculty of Biology, University of Freiburg, Freiburg, Germany; 5 BIOSS–Centre for Biological Signalling Studies, University of Freiburg, Freiburg, Germany; Chang Gung University, TAIWAN

## Abstract

Nested PCR remains a labor-intensive and error-prone biomolecular analysis. Laboratory workflow automation by precise control of minute liquid volumes in centrifugal microfluidic Lab-on-a-Chip systems holds great potential for such applications. However, the majority of these systems require costly custom-made processing devices. Our idea is to augment a standard laboratory device, here a centrifugal real-time PCR thermocycler, with inbuilt liquid handling capabilities for automation. We have developed a microfluidic disk segment enabling an automated nested real-time PCR assay for identification of common European animal groups adapted to forensic standards. For the first time we utilize a novel combination of fluidic elements, including pre-storage of reagents, to automate the assay at constant rotational frequency of an off-the-shelf thermocycler. It provides a universal duplex pre-amplification of short fragments of the mitochondrial 12S rRNA and cytochrome b genes, animal-group-specific main-amplifications, and melting curve analysis for differentiation. The system was characterized with respect to assay sensitivity, specificity, risk of cross-contamination, and detection of minor components in mixtures. 92.2% of the performed tests were recognized as fluidically failure-free sample handling and used for evaluation. Altogether, augmentation of the standard real-time thermocycler with a self-contained centrifugal microfluidic disk segment resulted in an accelerated and automated analysis reducing hands-on time, and circumventing the risk of contamination associated with regular nested PCR protocols.

## Introduction

The analysis of DNA by polymerase chain reaction (PCR) is a routinely applied method within most molecular biology laboratories, including forensic laboratories [1,2]. A wide range of target DNA is amplified for direct analysis or downstream applications [3]. Refining PCR strategies, as *e*.*g*. nested, inverse or touchdown-PCR have evolved dramatically over the years, increasing the specificity and sensitivity of DNA detection. Especially within the fields of investigative genetics as forensic science and food analysis, the analysis of human and animal DNA is required and samples with degraded or a low amount of DNA are a commonly occurring problem to overcome [2]. Species or animal family determination is an important application within the field of forensic genetics mainly *e*.*g*. to ascertain animals as perpetrators or for the investigation of illegal hunting [4]. Additionally, it is needed for analysis of (un-)declared ingredients within food. Mostly, restriction fragment analysis, sequencing methods, or probe-based species-specific real-time PCR are applied for analyses [5–9]. Currently, analysis of the PCR fragments by melt curve analysis and high resolution melting is increasing [10–13].

Sample mixtures and/or low copy number DNA can be successfully detected, *e*.*g*. by the use of nested PCR: A pre-amplified PCR product surrounding the region of interest is further amplified with another pair of primers within this PCR fragment in a second round of PCR. The final amplimers are then detected by gel or melt curve analysis. Time-consuming and error-prone manual pipetting work and a high risk of contamination, while handling high DNA copy numbers in-between PCR pre- and main-amplification, are major drawbacks of this method and may lead to false results.

While high-throughput laboratories reduce manual work by employing *e*.*g*. liquid handling robots, which facilitate the pipetting workflow in a fully automated fashion and reduce the risk of contamination and errors, the manual workflow remains the standard at low- to medium-throughput laboratories since the required equipment is expensive, less flexible and only well-suited for standard procedures.

Microfluidic Lab-on-a-Chip (LOC) systems aim to bridge this gap by bringing automation of laboratory workflows in a miniaturized format at low costs to all sizes of laboratories [14]. An especially attractive type of LOC systems, termed Microfluidic Apps, have evolved for seamless integration into existing instrumentation and workflows at low required financial investment: By coping with the constraints of standard laboratory instruments as processing devices, Microfluidic Apps may augment them with a process automation via liquid handling [15]. A prominent standard laboratory instrument for PCR is the Rotor-Gene Q (RGQ, QIAGEN, Hilden, Germany), featuring air-based thermal cycling of a rotary carousel and fluorescence signal detection. The axis of rotation of the RGQ thereby constitutes a common actuator for LOC systems, which apply centrifugal forces [16]. In this field of centrifugal microfluidics, the rotation is utilized to move liquid reagents *e*.*g*. of a biochemical assay from the center of a microfluidic disk to its outer rim, thereby forcing the liquid(s) to undergo all operations *e*.*g*. mixing, splitting, aliquoting that are required by the original, manual workflow [17]. While the rotation by itself causes centrifugal pressure inside the liquids *e*.*g*. for their transportation, a change in rotational speed is commonly necessary to open valves, which allow liquid(s) to move from one to the next operation [16]. In the RGQ, a change in rotational speed is not provided, but only rotation at low and constant 400 revolutions per minute (RPM). In addition, thermal actuation can only be executed under rotation [18]. The majority of known valving concepts on centrifugal microfluidic platforms do not cope with the constraints of the RGQ, as they require an increase from a lower to a higher rotational speed [19–21], a decrease from an initial high rotational speed [22], or active actuators for local heating [23–25] or cooling [26]. The capillary siphon valve [27,28] constitutes one of the rare embodiments that comply with the constraints of the RGQ and valve at a temporary state of low or zero rotational speed *e*.*g*. in-between consecutive (PCR) runs. Time-dependent valves [29,30] have the potential to operate at a constant rotational speed with the major drawback of having a hard-coded valving point in time. To allow context-based valving at constant rotational speed, we previously demonstrated the centrifugo-thermopneumatic fluid control, which is actively triggered by a global temperature change of the RGQ [31].

For the first time, we present a centrifugal microfluidic disk segment as Microfluidic App on an off-the-shelf RGQ that automates an assay for animal family determination by nested PCR and subsequent melt curve analysis to face problems as (cross-)contamination, error-prone pipetting and higher costs of common manual workflows. The assay was previously demonstrated as a conventional nested PCR, consisting of a universal pre-amplification and animal-group-specific main-amplification of two mitochondrial gene parts [11]. Here, adaptions and extensions of the assay allow a full integration of all required reagents into the Microfluidic App, which augments the RGQ to perform all liquid handling steps for the nested PCR protocol utilizing tailored centrifugal microfluidics instead of modifying the Rotor-Gene [32–35]. In turn, identification of twelve different animal groups (Equidae, Phasianidae, Felidae, Cervidae, Canidae, Mustelidae, Leporidae, Caprinae, Bovini, *Sus scrofa*, human and rodents (mouse and squirrel)) including all required controls was automatically performed to examine sensitivity, specificity and fluidic reliability of the system. Finally, case samples of forensic investigations were analyzed to test the applicability for routine applications.

## Materials and Methods

### Screening assay for identification of animal groups

The nested PCR assay detects parts of the mitochondrial genes encoding 12S rRNA and cytochrome b (cytb). It consists of a universal pre-amplification and animal-group-specific main-amplification. A previous version was published for the conventional use in tubes [11]. Along with microfluidic adaptions, the assay was also extended to additionally test for common representatives of the subfamily Caprinae (sheep, goat) and the order Rodentia (mouse, squirrel). Thus, twelve different animal groups can be discriminated. An animal group was successfully determined, if the two melting peaks representing the *12S rRNA* and *cytb* PCR products were clearly identifiable and discriminable from the background. Assay primers and concentrations used are stated in S1 Table.

### Adaptions of assay for implementation in microfluidic disk segment

Compared to the tube-based assay, the following adaptions were required:

1. Pre-storage of reagents: Liquid PCR master mix was replaced by lyophilisates (including DNA polymerase, dNTPs and EvaGreen dye as major components), air-dried reagents and a stabilizing sugar [36] (*cf*. Fabrication and S1 Table).
2. Nested PCR omitting intermediate purification: Prolonged (tagged) animal-group-specific primers allowed for a higher annealing temperature during main-amplification. Binding of the pre-amplification primers was thereby prevented, which circumvents the purification of the pre-amplification PCR product of the tube-based workflow. The tags added also allowed for verification by sequencing of the obtained PCR products (*cf*. Specificity experiments). The insertion of inosine into the universal *cytb* pre-amplification primer facilitated annealing to the target sequence more divergent between the animal groups and further destabilized the primer binding at the high temperatures of the main-amplification.
3. Shift from block cycler to centrifugal RGQ thermocycler: Primer concentrations and the PCR temperature protocol were adapted to the increased heating and cooling rates of the RGQ compared to the AB7500 (with 7500 System SDS software v1.4, Life Technologies, Carlsbad, CA, USA). Changing from a continuous melt process (AB7500) to a step-and-hold mechanism (RGQ) shifts the observed melting temperatures (T_m_) [37], which had to be considered.

### Design of microfluidic disk segment

**Fig 1:**
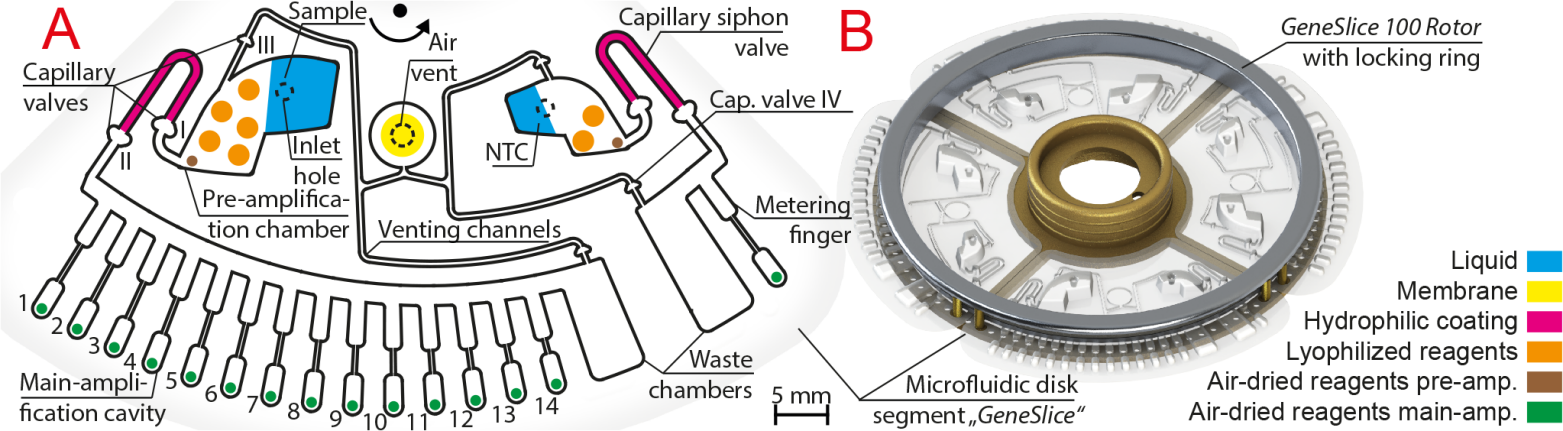
Schematic description of a microfluidic disk segment (“*GeneSlice”*) with pre-stored reagents (A), and a compatible rotor holder (“*GeneSlice 100 Rotor”*), which can hold up to four *GeneSlices* in one Rotor-Gene Q run (B). The *GeneSlice* comprises pre-amplification chambers for sample and no-template control (NTC) liquids, capillary siphon valves for transfer of the pre-amplification product and a centrifugo-thermopneumatic two-stage aliquoting structure [31] for 14 (sample) and 1 (NTC) main-amplification(s), respectively. The aliquoting structures guide excess liquid into waste chambers. All required reagents are pre-stored in lyophilized or air-dried format. The air vent for pressure equalization is covered with a membrane. Siphon valves are rendered hydrophilic by coatings.

The RGQ takes up rotors with tubes, which can either be spaced in 10°, 5°, or 3.6° angular distances. A custom rotor holder (*GeneSlice 100 Rotor*) was designed (*cf*. Abstract Graphic), which provides pins to precisely align reaction cavities of the microfluidic disk segment, termed *GeneSlice*, to the stationary fluorescence detector of the RGQ while passing the detector under rotation (Fig 1B). The spacing of reaction cavities was set to 3.6° to accommodate a maximum of parallel PCR reactions. The *GeneSlice* (Fig 1A) consists of two fluidic networks of chambers and channels fluidically processing one sample (left side) and the corresponding no-template control (NTC; right side) during nested PCR and subsequent melt curve analysis.

The two fluidic paths are symmetrically arranged on the *GeneSlice* and share a network of venting channels towards the middle, which ends at an air vent that allows pressure equalization during thermal cycling. Each fluidic path includes three major structures performing basic (unit) operations that are scaled to the volumes of the sample and NTC liquid, respectively: (1) a pre-amplification chamber with pre-stored lyophilized and air-dried reagents, which allows liquid insertion through a sealable inlet hole [34] into a dedicated loading area, (2) a capillary siphon valve, which transfers the pre-amplification product into a downstream (3) centrifugo-thermopneumatic (CTP) two-stage aliquoting structure [31]. In addition, capillary valves (Fig 1A, I) prevent premature siphon valving during handling of the *GeneSlice*, (Fig 1A, II) premature capillary filling of the aliquoting structure, and (Fig 1A, III and IV) unintended capillary priming of venting channels.

### Fabrication of the microfluidic disk segment and pre-storage of reagents

The *GeneSlice* was designed in SolidWorks 2011 (Dassault Systèmes SolidWorks, Waltham, MA, USA) as computer-aided design (CAD) and manufactured in-house by the Hahn-Schickard Lab-on-a-Chip Design + Foundry Service (Hahn-Schickard, Freiburg, Germany) applying a μTSL replication process [34]. The capillary siphon valve of the sample (and NTC) fluidic path was rendered hydrophilic by pipetting a hydrophilic coating into the region in-between capillary valves (Fig 1A, I and II). Reagent mixes for the sample and NTC pre-amplification including universal *12S rRNA* and *cytb* primers, trehalose as long-term stabilizer [36], and ROX as passive reference dye were prepared and pre-stored into the sample and NTC pre-amplification chambers (*cf*. Fig 1A). If not stated otherwise, the main-amplification cavities (*cf*. Fig 1A) were pre-stored with trehalose, EvaGreen, and animal-group-specific primers (cavity 1–12 for the twelve animal groups), universal *12S rRNA* primers as extraction control (cavity 13), and with an 81 bp long oligonucleotide with specific primers as internal positive control (cavity 14) as given in S1 Table. Details on the fabrication process and pre-stored reagents can be found in the experimental details (S1Text).

For pre-storage of polymerase, dNTPs and buffer ions, five and two lyophilized PCR beads (qPCR GreenMaster Lyophilisate, PCR-157L-FTP, Jena Bioscience) were transferred by a pick-and-place process into the sample and NTC pre-amplification chamber, respectively (*cf*. Fig 1A). Subsequently, the microfluidic disks were sealed, covering the open side of the micro-thermoformed film with a pressure-sensitive adhesive polyolefin foil (#900 320, HJ-Bioanalytik, Erkelenz, Germany). The sealing foil included laser-cut (PLS3.60, Universal Laser Systems, Scottsdale, AZ, USA) air vent holes, each covered with a membrane (FGLP01300, Merck, Darmstadt, Germany) to highly reduce the risk of contamination by potential aerosols. The sealed microfluidic disk was laser-cut into *GeneSlices*, each including two laser-cut holes for correct positioning on the *GeneSlice 100 Rotor* (Fig 1B). *GeneSlices* were finally packaged in aluminum barrier film flat bags (A 20T, Long Life for Art, Eichstetten, Germany) with silica gel desiccants (#610.002, ThoMar, Luetau, Germany) under nitrogen atmosphere to protect the pre-stored reagents until usage.

### General setup

Pre-amplification of the sample (template DNA, 10μg of bovine serum albumin (BSA, Roche, Basel, Switzerland), PCR-grade water) and the NTC (4μg BSA, PCR-grade water) was carried out at reaction volumes of 100μL and 40μL, respectively, which were pipetted into the corresponding inlet holes (Fig 1A). A lower NTC volume was chosen to decrease reagent consumption and dilution effects of contaminants within the pre-amplification volume. Afterwards, inlet holes were sealed with adhesive tape (#95.1994, Sarstedt, Nuembrecht, Germany). Up to four *GeneSlices* per run were inserted into the *GeneSlice 100 Rotor* and were secured with a locking ring (Fig 1B). For maximum temperature uniformity and to ensure an even airflow to every reaction cavity [18], empty slots on the *GeneSlice 100 Rotor* were filled with empty *GeneSlices*. The assembly was then inserted into the RGQ chamber, similar to standard tube rotors (*cf*. Abstract Graphic). All temperature steps for PCR, aliquoting and DNA melting as well as data evaluation were conducted using the RGQ Series Software (version 2.2.3 build 11, QIAGEN).

After insertion, the pre-amplification run was started creating a first run-file. Then, a custom monitoring script based on AutoIt (v3, AutoIt Consulting, England, script will be provided upon request) was initiated and provided with required information on the main-amplification run by the operator creating a second run-file, before walking away from the system. The monitoring script autonomously registered the end of the pre-amplification run and subsequently started the main-amplification run after a defined and short period of resting.

### Microfluidic functional principle and protocols

**Fig 2:**
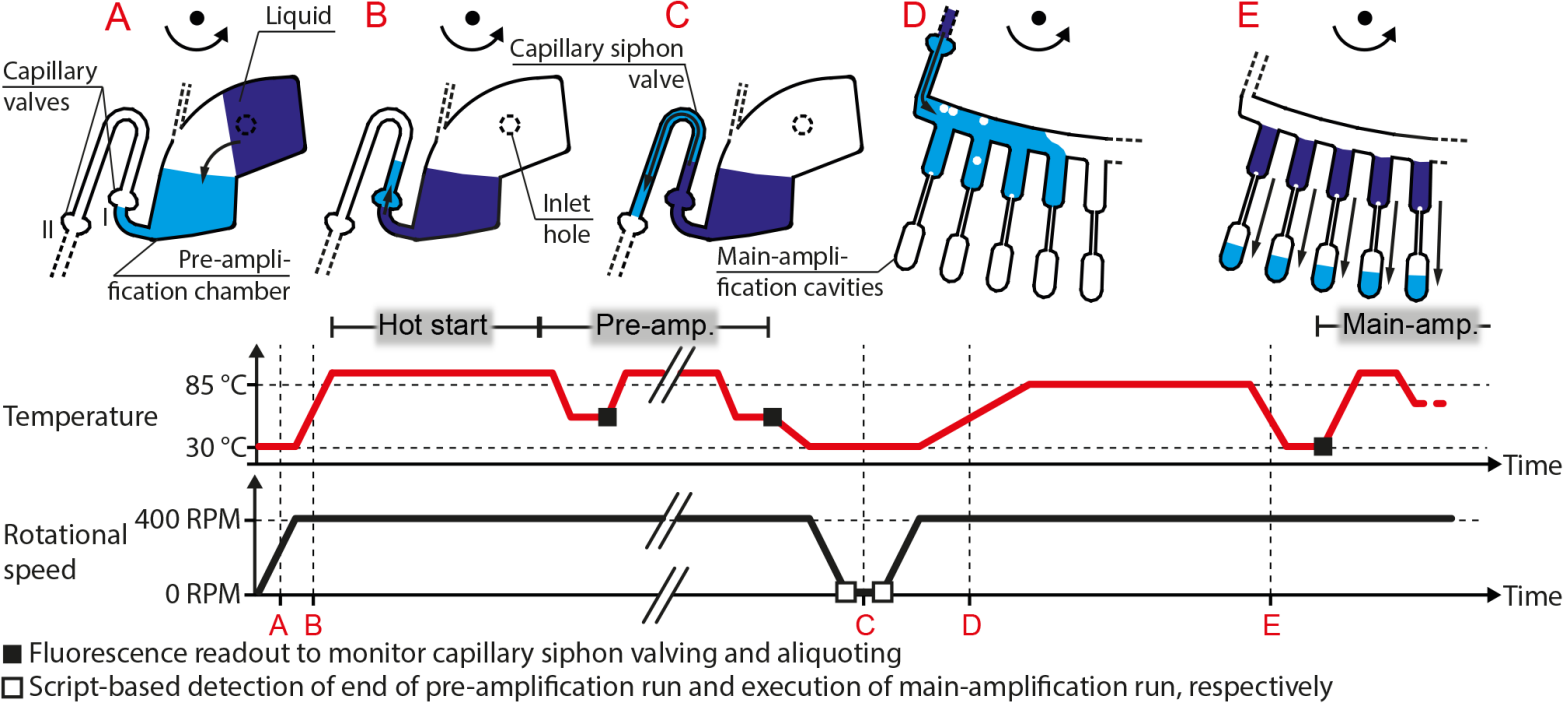
Illustration of microfluidic process flow of a *GeneSlice* for nested PCR and melt curve analysis on a Rotor-Gene Q. The microfluidic structures of interest at different points in time (A-E) are shown on top, indicating liquid movement from the dark blue to the light blue position. Corresponding temperatures (red) and rotational speed (black) at each depicted point in time can be read from the given diagram at the bottom. The liquid stays in the position depicted in B during pre-amplification, which consists of 10 thermal PCR cycles at constant 400 RPM. During main-amplification and melt curve analysis, which follow the last (E) depicted fluidic operation, no further fluidic operations take place and liquids stay in the main-amplification cavities (E, light blue).

Starting the pre-amplification, the loaded sample/NTC liquids are transferred from their loading areas into the radially most outward position of the pre-amplification chambers by constant centrifugation of 400 RPM (Fig 2A). The first capillary valves (Fig 2, I) directly break by the centrifugal pressure of the sample/NTC liquid. Breaking of the valves is enhanced by the lowered surface tension and degassing of liquid, which pushes liquid through the valve. Both is caused by the temperature ramp up to the hot start temperature of the polymerase (Fig 2B). During further constant centrifugation, the liquids stay in the pre-amplification chambers, and pre-amplification of 10 PCR cycles is carried out. Afterward, a programmed cool-down to 30°C takes place (hold at 40°C for 0 s, at 35°C for 0 s, at 30°C for 40 s), before the RGQ shuts down resulting in a cool-down to room temperature and a subsequent halt of rotation. During this resting period, the capillary pressures inside the hydrophilic siphon valves pull the pre-amplification products beyond the radially inward siphon’s crests to the capillary valves (Fig 2, II), which stop further capillary priming (Fig 2C) [17]. When the main-amplification is initiated, the rotor accelerates to 400 RPM and the centrifugal pressures break the capillary valves (Fig 2, II). The liquids are propelled downstream into the corresponding CTP two-stage aliquoting structures.

In brief, for CTP two-stage aliquoting temperature is increased from 40 to 85°C (2°C 1 s^-1^) and held for 6 min under rotation. Centrifugation leads to metering of sample pre-amplification product into 14 sub-volumes by filling metering fingers, each serving as an overflow vessel to the next one (Fig 2D). Temperature increase thereby increases pressure inside the main-amplification cavities, which prevents the metered liquids from penetrating them: Pressure increase causes gas bubbles to leave the reaction cavities (Fig 2D and 2E), which in turn constantly push back and hold the liquid sub-volumes in their corresponding metering fingers. After the metering process, the temperature is lowered to 30°C and held for 2 min, which causes volume contraction of the gas inside the main-amplification cavities. Consequently, aliquots are sucked into the reaction cavities for main-amplification and melt curve analysis (Fig 2E). Simultaneously, NTC pre-amplification product is aliquoted into a single main-amplification cavity in an analog manner. All the temperature steps required are included in the main-amplification run file as created by the operator. CTP aliquoting is solely controlled by temperature and operates at the constant rotational speed of the RGQ. Detailed description of the underlying physical principle will be available soon (manuscript in preparation).

### Failure alert mechanism

In all experiments, fluorescence readouts at all main-amplification cavities on the orange channel (ROX) (excitation at 585 nm and detection at 610 nm) were carried out at the end of each annealing step of pre-amplification as well as the cool-down to 30°C of the initial aliquoting temperature profile in the main-amplification run (Fig 2). The examination of the acquired signals served as failure alert mechanism: No signal increase during pre-amplification and an increased signal after aliquoting was expected in fluidicially fully functional *GeneSlices*. In case of premature siphon valving during pre-amplification, a signal increase during pre-amplification was expected to give information on the affected *GeneSlice* and structure (sample or NTC) and the cycle of occurrence. If siphon valving did not take place after pre-amplification, missing signal increase(s) after aliquoting was expected to indicate the affected *GeneSlice*, structure and main-amplification cavity/-ies. The examination was carried out as a follow-up to the runs.

### Setup for fluidic characterization

Fluidic characterization of the *GeneSlice* was performed without DNA utilizing a RGQ, which was modified for inside stroboscopic image acquisition (BioFluidix, Freiburg, Germany), termed strobe RGQ (S1 Fig). Stationary images of the rotating *GeneSlices* at arbitrary azimuthal position were thereby acquired (S1 Video), which enabled qualitative analysis of liquid movements and quantitative post-processing of the recorded images: For quantification of the aliquoted volumes, the filling heights of the liquid inside of each main-amplification cavity (sample structure) was measured with ImageJ (National Institutes of Health, Bethesda, MD, USA), and volumes were calculated in SolidWorks feeding the measured values into the CAD.

### Thermal protocol for nested PCR

Pre-amplification was carried out with an initial hot start of the polymerase and DNA denaturation at 95°C for 5 min, and 10 cycles with the machines ramp rates [18] of 45 s at 95°C and 75 s at 56°C. Main-amplification cycling was divided into two sections for improved primer annealing: 6 cycles of 45 s at 95°C and 80 s at 68°C, followed by 20 cycles of 45 s at 95°C and 80 s at 73°C. 10 min at 73°C were used for a terminal elongation. For DNA melting, temperature was ramped from 68–88°C with 0.5°C/step and 5 s hold and an initial denaturation of the PCR products at 95°C for 45 s. Fluorescence was acquired within the green (excitation at 470 nm and detection at 510 nm) and orange channels during pre- and main-amplification and the green channel during DNA melting.

### Tests for determination of specificity and sensitivity

Tissue material was obtained from the Department of Animal Hygiene, CVUA, Freiburg, Germany. DNA was extracted from muscle samples, if available. Bovine and donkey DNA was obtained from blood and buccal cells, respectively. Each cell type was extracted as described elsewhere [13]. For some species DNA, extracts were directly obtained from the Institute of Veterinary Pathology, University of Giessen, Germany. Sample origin was verified by sequencing as described [11]. Tissue sampling and DNA analyses were approved by the Ethics Committee of the University of Freiburg (Approval Number: 419/09_130559). For the majority of animal groups, 1 ng of template DNA from two different species per group was tested, except for the “groups”, Bovini (*Bos taurus*), *Sus scrofa* and *Homo sapiens*, using one *GeneSlice* for each species. Additionally, DNA of following species not covered by the assay was tested to exclude cross-reactivity: macaque, budgerigar, pogona, and carp. Besides visual interpretation of melt curves, products of the animal-group-specific amplification of the *12S rRNA* and *cytb* gene fragments, as well as the universal *12S rRNA* amplimers were sequenced for verification using the primer tag as sequencing primer (*cf*. S1 Table). In addition, PCR products of reaction cavities showing (an) unintended peak(s) in their melt curve were sequenced for clarification. For sensitivity level determination, the needed template amount for a clearly detectable amplification of one species within each animal group was contained, testing 20, 50, 100 or 200 pg DNA. Samples from two species were then combined on one *GeneSlice* and analysis was repeated three times for reproducibility. The second species of each animal group, which was also used for specificity experiments, was analyzed once using the same amount of DNA as the first one, if possible, or with an increased template amount if necessary for a better ranking of the sensitivity limits within each group.

### Detection of minor components within DNA mixtures

Two series of experiments were conducted: First, the ratio of pig and human was altered from 0% to 100% in a mixture, with each ratio tested once. Secondly, a mixture of human/ pig/ cow/ roe deer with one main contributor was tested with a total of three repetitions. Here, the detection level of each component within a mixture of 1 ng total DNA was first determined (50 pg (5%) for pig, human, and cow DNA and 100 pg (10%) for roe deer DNA), and then additionally repeated twice. For both series, the pre-storage pattern in the main-amplification cavities was changed during fabrication to the following: To detect the human/ pig mixture, human specific, pig specific, and the universal primer pairs were pre-stored as triplicates in three and the IPC as duplicate in two consecutive reaction cavities, respectively. To test cow/ human/ pig/ roe deer mixtures, triplicates of human-, pig-, Bovini- and Cervidae-specific primers were pre-stored in consecutive reaction cavities, respectively.

### Examination of risk of cross-contamination

The risk of potential cross-contamination during the main-amplification, which is highly reduced by two-stage compared to one-stage aliquoting [20] was nevertheless examined similarly to [34] as the reaction volumes are not hermetically isolated from each other. The examination required a different pre-storage pattern during fabrication: A DNA sample from red deer (1 ng template DNA) and an NTC control were pre-amplified in tube for 10 cycles. 1 ng DNA corresponds to around 150 cells with *e*.*g*. 3650 ± 620 mtDNA copies/cell within skeletal muscle in human [38]. Accordingly, it was roughly calculated to have 560 x 10^6^ ± 95 x 10^6^ copies after a pre-amplification with 100% efficiency. For pre-storage, 7μL of the pre-amplification product (corresponding to approx. 39.2 x 10^6^ ± 6.65 x 10^6^ copies) was given in every second main-amplification cavity. Likewise, 7μL of the 40μL NTC main-amplification was pre-stored in the NTC main-amplification cavity. The lyophilized beads were rehydrated by the insertion of Cervidae-specific primers (150 nM *12S rRNA* and 200 nM *cytb*) combined with BSA (14μg) and PCR-grade water (*ad* 140μL) into the sample (100μL) and NTC (40μL) pre-amplification chambers. In the pre-amplification run, thermal cycling was skipped performing only the 5 min at 95°C hot start and the controlled cool down to 30°C. Subsequent to aliquoting, during main-amplification and melt curve analysis, Cervidae-specific amplification signals and peaks were expected in reaction cavities with pre-stored pre-amplification product only. The experiment was performed in four *GeneSlice*s.

### Forensic case studies

A bone sample (spine), dried meat from Namibia (Biltong meat declared as “Game meat”), a piece of fabric from a tent (saliva/skin cells), and a swab (smear from a jacket) were tested to prove applicability of the *GeneSlice* to forensic case work. DNA of the bone and meat was extracted as described elsewhere [39], and the fabric and swab by using the MN Tissue Kit (MACHEREY-NAGEL, Dueren, Germany) according to the manufacturer’s protocol. The bone sample (found in a local forest) and meat sample (closed food package) were distinct pieces without any morphological sign of a mixed origin. As reference, a universal PCR and sequencing analysis of the longer fragments (~400 and 480 bp, respectively) of *12S rRNA* and *cytb* has been performed [11]. The fabric sample was part of an investigation of an animal attack published earlier [40]. In brief, species identification of the perpetrator was needed. A piece of a tent fabric was cut from the location of a bite mark, expecting a mixture from the human victim and the attacking animal. This was confirmed by the reference universal sequencing approach detecting a non-human sequence component leading to further investigation by cloning as well as group- and species-specific PCR for mixture resolution [40]. 1 ng of extracted DNA was used in case of the bone, meat and fabric sample for analyses in *GeneSlices*. The swab obtained from a jacket tested positive in a presumptive blood test was part of a murder investigation. Due to a low DNA concentration and limited sample amount, only 350 pg of DNA were available for re-analysis on the *GeneSlice*. Originally, a sequencing reaction as mentioned above as well as manually handled animal-group-specific PCR, melt curve analysis and sequencing for confirmation had been performed. Primers for the reference methods can be found in [11,40] and S1 Table.

## Results and Discussion

### Failure alert mechanism

A failure alert mechanism was implemented by fluorescence readout signals of main-amplification cavities of all processed *GeneSlices* (n = 102, the four *GeneSlice* runs for examination of cross-contamination risk where not included). All cases of premature siphon valving and cases where valving did not take place were thereby successfully detected (S2 Fig).

In six (5.9%) and 13 cases (12.7%) sample and NTC liquid, respectively, prematurely valved during pre-amplification, all but one within the first pre-amplification PCR cycle. Upon detection of the failure “premature valving” of sample liquid, corresponding experiments were repeated. Premature valving of NTC liquid did not require repetition of experiments, as it was expected not to affect the detection of potential contaminants: Reactions may have continued similarly within the main-amplification cavity and were not biased by the reagents within this cavity, as they also included universal primers. In addition, contaminations would have been noticed by melt peaks within unexpected cavities of the sample structure, as the species of the DNA included in the reaction was known. Prevention of premature valving is discussed in the Fluidic characterization section. In two (2.0%) and 24 cases (23.5%) siphon valving did not take place for sample and NTC liquid, respectively. Further investigation showed that elongated waiting time between the pre- and main-amplification runs allowed for capillary siphon valving in the majority of cases (*cf*. Fluidic characterization). Thus, the fluorescence readout signals indicating that valving did not take place may be utilized in the future to monitor capillary siphon valving by another programmed monitoring script, which may set the RGQ to shut-down and wait before re-starting the main-amplification run again. There were no false-negative results caused by fluidic malfunction at any time of the study. The monitoring script worked properly in all cases, detecting the end of the pre-amplification and starting the main-amplification run after 18.0 ± 0.0 s. In 94 cases (92.2%) of this study, the fluidics of the *GeneSlices* proved fully functional for the sample structure, disregarding the NTC.

### Fluidic characterization

In seven out of 19 cases with premature valving, a small liquid plug inside the region of the venting channel in-between the pre-amplification chamber and the capillary valve (Fig 1A, III) was observed with naked eye by the operator prior to execution of the test, and may have been present in all cases. The strobe RGQ revealed that this unintended liquid plug fails to be spun from the venting channel back into the pre-amplification chamber before heating for hot-start is initiated. This in turn expands the enclosed air volume inside the pre-amplification chamber (S3 Fig), and pushes liquid through the siphon valve within the first cycle. In the majority of cases liquid was unintentionally brought into contact with the venting channel, caused by the operator’s pipetting and/or by the rehydration process of the lyophilized beads. During the development of the *GeneSlice* when only liquid reagents were applied, this problem had not been encountered. The increased number of “premature valving” incidents that occurred in NTC compared to sample structures is likely caused by the closer proximity of the venting channel to the loading area of the NTC than the sample liquid. It is expected that by redesigning the venting channels and by pre-storing the lyophilized beads away from the loading area premature valving may be significantly reduced.

While highly concentrated Vistex coatings proved good reliability for capillary siphon valving [35], a much lower Vistex concentration had to be applied in this study in order not to outbalance the low centrifugal pressure induced by the RGQ during pre-amplification (*cf*. S1 Text). Accordingly, investigations on the strobe RGQ showed that siphon valving times may be higher than set in the monitoring script (18 s) in some cases, especially when transferring the minute NTC volume from its small pre-amplification chamber, which poses a counteracting capillary pressure by itself. It is assumed that this circumstance increased variance in valving times, which could, however, not be quantified as valving takes place at rest and does not permit image acquisition of the process. A redesign of the NTC pre-amplification chamber towards a reduced capillary pressure and the described autonomous script-based repetition of waiting time in-between pre- and main-amplification runs (*cf*. Failure alert mechanism), may accelerate and ensure valving even at slow propagation of liquid inside the NTC capillary siphon valve.

In addition characterization of aliquoting revealed a mean aliquoted volume of 4.5μL, an inter-*GeneSlice* coefficient of variation (CV) of 2.0% and a mean intra-*GeneSlice* CV of 6.3% (n = 8 *GeneSlices*).

### Assay specificity and sensitivity

**Fig 3:**
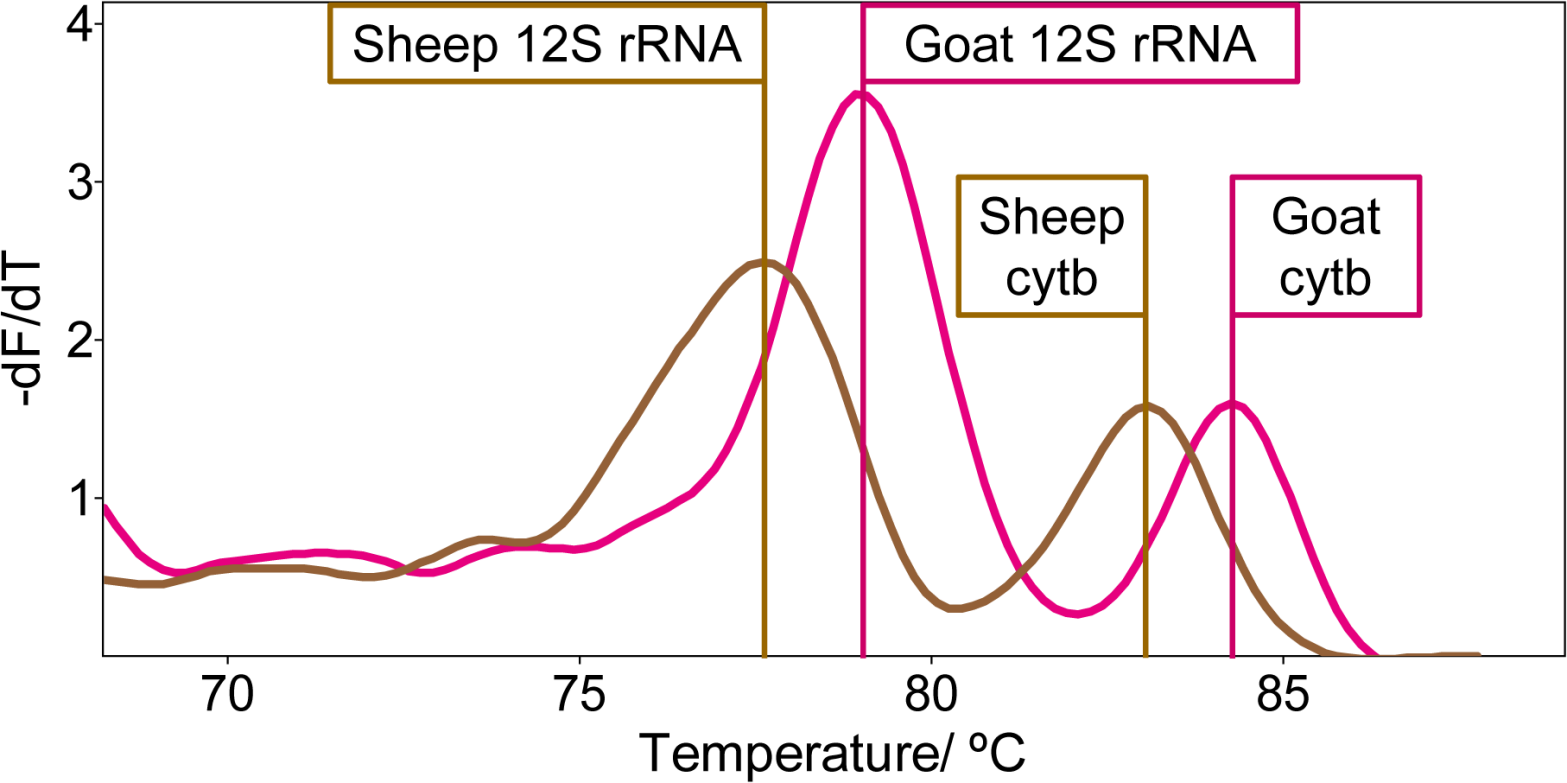
Melt curve analysis of sheep and goat amplimers. 1 ng of sheep and goat DNA were amplified on two *GeneSlices*. The melt curve analysis shows the successful amplification of the *12S rRNA* and *cytb* genes for both species. The different melting behavior is caused by inter-species sequence differences within the Caprinae.

1 ng of DNA from two species of each animal group, except for Bovini, *Homo sapiens* and *Sus scrofa*, were used for verification of the animal-group-specific primers. A successful amplification was obtained in all cases by detection of the intended melt peaks for both mtDNA fragments (*12S rRNA* and *cytb*), as depicted for *e*.*g*. Caprinae in Fig 3. Macaque and budgerigar, not covered by the assay, only resulted in a positive amplification of the universal *12S rRNA* fragment (DNA extraction control), whereas no signal was obtained for pogona and carp due to an insufficient amplification efficiency.

However, unexpected signals were occasionally observed within other main-amplification cavities. The respective PCR products were sequenced to examine these unexpected melt peaks. The expected sequences were obtained for both the specific *12S rRNA*/ *cytb* and the universal *12S rRNA* primer pairs. Nuclear mitochondrial DNA of cow and cat was co-amplified by the universal *12S rRNA* primer pair without compromising data interpretation. In addition, sequencing of unexpected peaks revealed a residual activity, especially of the pre-amplification *12S rRNA* forward and *cytb* reverse primers slightly decreasing the test’s specificity by their universal character. Non-specific profiles can however be discriminated due to their shifted melt peaks. Thus, only signals of both, *12S rRNA* and *cytb* targets, within the expected temperature range are recommended as a prerequisite for a positive animal group call. S2 Table contains the species specific melting temperatures (T_m_) obtained from the specificity and sensitivity experiments. To test the robustness of successful amplification with low amounts of DNA, a determination of the detection limit down to 20 pg of one representative of each group revealed positive signals for the majority of groups (Table 1). Unsuccessful amplifications were repeated with an increased amount of DNA, obtaining positive signals for all species tested within a range of 20 and 200 pg. Three repetitions of runs revealed a good robustness of the system without any failure of amplification due to stochastic effects (*cf*. coefficients of variation in S2 Table). The analysis of a second species mostly revealed the same detection capacity except for lynx, donkey and weasel, which required an increased amount of DNA. It has to be considered that in case of donkey buccal cells with a lower content of mtDNA than muscle were used as DNA source. In addition, differences in sensitivity within animal groups can occur due to the sequence differences in-between species at the primer binding position altering the efficiency of primer annealing. Nevertheless, the level of sensitivity was mostly restricted by the amplification of *cytb*. A less efficient pre-amplification due to higher sequence diversities at the primer binding site and the higher GC content probably caused a PCR bias in favor of *12S rRNA*. An increase of the universal *cytb* primer amount during pre-amplification was not pursued in order to prevent undesirable binding of residual universal primers during the main-amplification as much as possible.

**Table 1:** Sensitivity level of species analyzed within the assay for detection of European animal groups on *GeneSlices*. Bold: species analyzed in triplicates.

DNA amount	Species	
**20 pg**	**Dog**/ Fox	**Cat**
	**Rabbit**/ Hare	**Pig**
	**Goat**/ Sheep	**Horse**
	**Red deer**/ Roe deer	**Pine marten**
**50 pg**	**Human**	Weasel
**100 pg**	**Mouse**/ Squirrel	
**200 pg**	**Chicken**/ Turkey	Donkey
	**Cow**	Lynx

### Detection of minor components within mixtures

**Fig 4:**
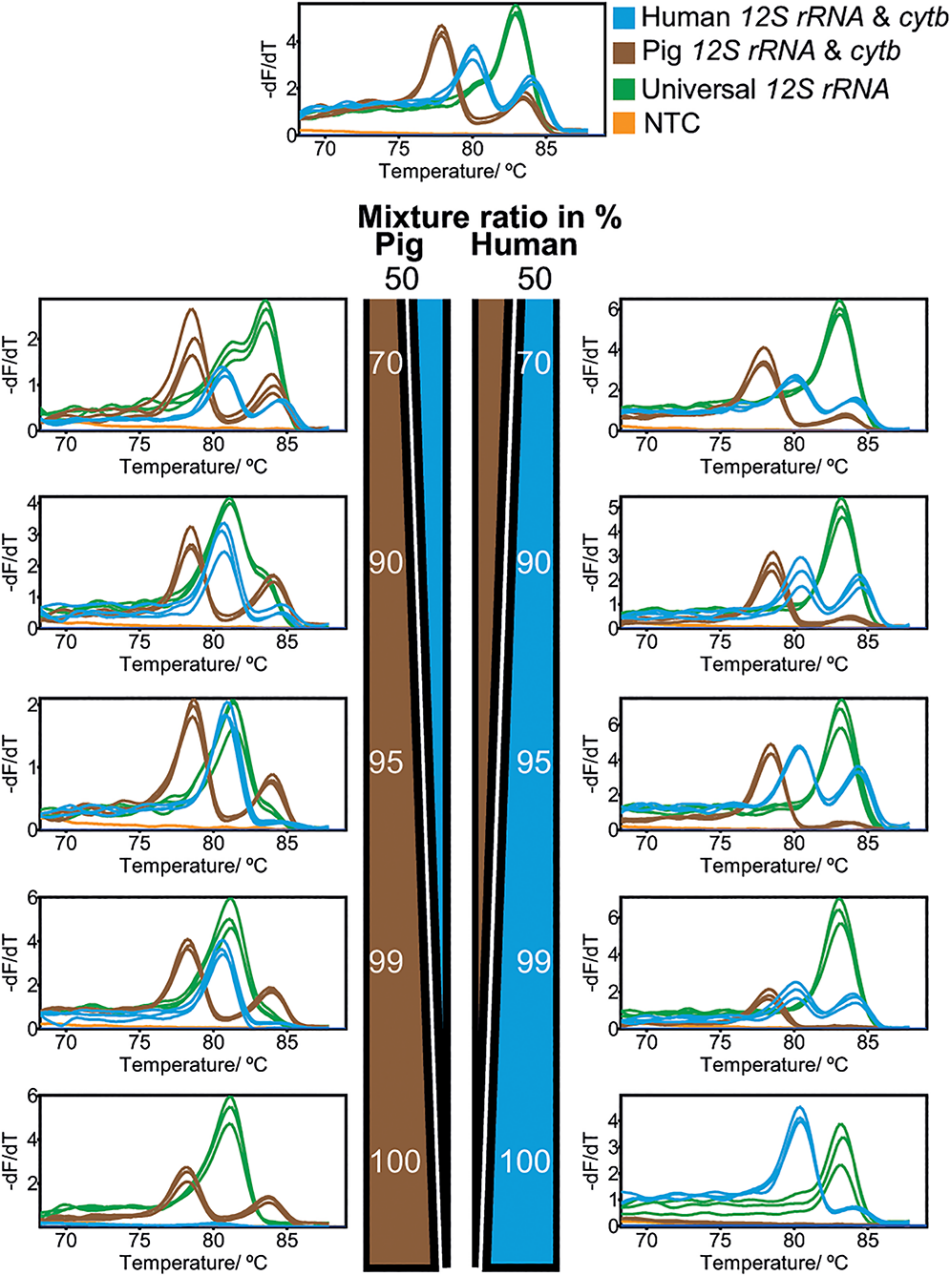
Melt curve analysis of a mixture of human and pig DNA in different ratios. Artificial mixtures of human and pig DNA were created from 0% to 100%, each, and amplified. Analysis of the respective animal-group-specific main-amplification and universal *12S rRNA* cavities shows resolution of the DNA components down to 1% (*12S rRNA*) for pig and human; 5% both for pig, and 10% both for human as minor components.

Mixing of human and pig DNA (1 ng in total) led to successful detection of human and pig down to 1% for *12S rRNA* as well as 5% for pig *cytb* and 10% for human *cytb* (Fig 4). In all cases, the included internal positive control (IPC) and the universal *12S rRNA* used as positive DNA control showed the expected results. The IPC showed a stable melting behavior with a melting temperature of 81.71 ± 0.23°C over all measurements. There were only small deviations within the triplicates, with a maximum ± 0.13°C shift of T_m_ for the visually detectable universal, human-specific and pig-specific melting peaks.

Resolution of mixed samples of multiple species (human, pig, cow and roe deer) was successful with a less sensitive detection of roe deer (threshold at 10%) and less clear detection of cow-specific peaks resulting from the higher limit of detection of 200 pg (see above). As already mentioned, preferential pre-amplification due to variant primer binding sites may result in a loss of a minor component during the pre-amplification. 50 pg (human, pig, cow) and 100 pg (roe deer) were repeatedly detected within all tested multiple compound mixtures with one major component.

### Examination of risk of cross-contamination

To exclude the risk of cross-contamination by evaporation of PCR products in neighboring main-amplification cavities, pre-amplification product of 1 ng red deer was pre-stored in every second main-amplification cavity and amplified with Cervidae-specific primers (present in all main-amplification cavities after aliquoting). No cross-contamination or contamination within the NTC was observed using four independent *GeneSlices* (*i*.*e*. 28 cavities). CTP two-stage aliquoting may thus be regarded suitable for highly sensitive (nested) PCR analysis.

### Forensic case studies

Different available forensic samples were tested covering a wide range of common case situations as *e*. *g*. pure and mixed samples of different common and rare materials. A bone sample, dried meat, a fabric sample from a tent [40], and a swab from a jacket were analyzed. The results of the originally performed analyses as well as the performance of the *GeneSlice* are summarized in S3 Table. The *GeneSlice* allowed for drawing the same conclusions in case of the bone (sheep DNA) and fabric sample (mixture of human, and fox DNA). The *GeneSlice* was advantageous especially for the fabric sample, as the resolution of a DNA mixture was possible without further investigation methods (S4 Fig).

The analysis of the Biltong meat sample in a *GeneSlice* excluded “game” (as known in Europe) as ingredient, which was indicated by the absence of signals within the main-amplification cavities for Cervidae as well as for Sus scrofa. Positive extraction/PCR control showed valid signals for the test, revealing the origin of the meat as a species not covered by the assay. The reference sequence analysis revealed the sample’s origin as kangaroo, being a member of the family Diprotodontia.

The *GeneSlice* analysis of the jacket’s swab led to the identification of a human and potential bovine DNA contributor. The original intense analysis revealed human, pig, cow, and chicken DNA after application of various methods (S3 Table). It can be concluded from the unsuccessful universal *12S rRNA* and *cytb* sequencing that the DNA of the sample was degraded and only allowed for short fragment amplification, which was induced by the animal-group-specific primers in tubes or the *GeneSlice*. Only a very low amount of DNA was available for the re-analysis on the *GeneSlice* after the original investigation, which may explain a “loss” of some of the minor components.

In sum, the *GeneSlice* results essentially matched the reference sequencing reactions (kangaroo excluded, which is not covered by the assay), and showed a better resolution of mixed DNA (tent sample). The pre-storage of reagents inside the *GeneSlice* and its closed reaction environment without further manual handling are decreasing the time required for processing as well as the risk of contamination. The one-step procedure, which results in raw data acquisition from fully processed melt curve analyses of all 15 (including the NTC) reaction cavities, may be regarded labor-effective compared to the several reaction steps needed for the standard PCR and sequencing (e.g. gel analysis, clean up steps and set-up of the sequencing reaction). Furthermore, time-consuming cloning techniques for resolution of mixture components are omitted. Acquired raw data was processed as usual using standard analysis functions of the RGQ Series Software. If signals were clearly differentiated from the background signals, calling of a peak allowed for unambiguous assessment of the forensic sample due to the given location of pre-stored primers. The *GeneSlice* may thus pose a well-applicable and advantageous tool in forensic routine case work.

## Conclusion

A forensic nested PCR assay with melt curve analysis covering twelve important European animal groups was successfully automated by a centrifugal microfluidic disk segment designated as “*GeneSlice”* that was processed by a standard laboratory real-time PCR thermocycler (Rotor-Gene). Although adaptions were required in comparison to the original assay performed by manual pipetting, specificity, sensitivity, and mixture analysis revealed comparable and reliable results and processing of case work samples confirmed the applicability of the *GeneSlice* in routine analysis. Especially the high contamination risk between the first and second round of PCR prevented application of manually performed nested PCRs in forensic laboratories. The *GeneSlice* augments standard Rotor-Genes to perform nested PCR with a minimum of pipetting steps, reduced reagent consumption and reduced risk of contamination and is regarded applicable to similar fields of applications. A redesign of the NTC pre-amplification chamber will reduce the possibility of handling errors during the fill-in process by the operator, in turn increasing reliability of the fluidic functionality (currently 63.7% for NTC), which proved to be at a high level for the sample with 92.2%. The demonstrated capabilities of script-based monitoring of the RGQ software to autonomously take pre-defined actions may be extended to monitor and ensure fail-safe operation. The *GeneSlice* will thus conduct a very reliable walk-away automation solution for nested PCR applications with inbuilt fail-safe operation throughout the analysis, which is manufactured in a scalable prototyping technology including pre-storage of all required reagents.

## Supporting Information

S1 Fig Strobe RGQ. The strobe RGQ allows acquisition of stationary images of the rotating *GeneSlices* at arbitrary azimuthal position. It consists of an add-on with a camera, which can be positioned in x- and y-direction. A 45 degree mirror allows observation of the *GeneSlices* from underneath.
(PDF)

S2 Fig Failure alert mechanism. Fluorescence signal increase during pre-amplification indicates that the corresponding main-amplification cavity was filled by premature siphon valving (left). If signals do not increase during aliquoting, it indicates that capillary siphon valving did not take place for the corresponding structure (right).
(PDF)

S3 Fig Explanation for premature valving. If a liquid plug is unintentionally present in the venting channel, an enclosed air volume (light red) is expanded during temperature increase of the hot start for PCR. As a result, the liquid (dark blue) is displaced through the siphon valve (light blue) during pre-amplification.
(PDF)

S4 Fig Melt curve analysis after PCR with a *GeneSlice* of DNA recovered from an animal’s bite mark on a tent. The analysis reveals the occurrence of human DNA and DNA from a species of the family of Canidae. Characterization of the melt peaks identified the DNA origin as fox, which matches an earlier conducted and published examination of the sample with additional species-specific PCR. Controls included in the *GeneSlice* showed valid signals.
(PDF)

S1 Table Primer sequences and amount used for pre-storage in the *GeneSlice*. Universal *12S rRNA* and *cytb* for pre-amplification were pre-stored within the Sample and NTC pre-amplification chambers. The *12S rRNA* and *cytb* animal-group-specific primers of the twelve groups, as well as the universal *12S rRNA* primer pair and internal positive control (IPC) consisting of a primer pair and an 81 bp oligonucleotide were pre-stored in the 14 main-amplification cavities of the sample. The universal *12S rRNA* primer pair was additionally used for the main-amplification cavitiy of the NTC.
(PDF)

S2 Table Melting temperatures (T_m_) of amplimers, as *e*.*g*. depicted in Fig 3 using different amounts of DNA. Left: T_m_ of amplification of 1 ng of genomic template DNA; Right: T_m_ of amplification of the minimal needed DNA amount for successful peak detection (*cf*. Table 1 for template amount), the coefficient of variation (CV) is given for one representative per group; as these were analyzed in triplicates.
(PDF)

S3 Table Summary of results of forensic case samples. Comparison between reference and *GeneSlice* analyses. T_m_: Melting temperature.
(PDF)

S1 Text Fabrication of the microfluidic disk segment and pre-storage of reagents. Detailed technical information about the processing steps and materials are presented.
(PDF)

S1 Video Stationary images of the rotating *GeneSlice* acquired by the strobe RGQ. The video shows the pre-amplification of the sample and its subsequent centrifugo-thermopneumatic aliquoting. The top left indicates the time in ms. The bottom left either indicates the temperatures of the RGQ software (modelled) or the rotational speed. The latter is falsified by the acquisition mode (multi-trigger) of the strobe RGQ (four *GeneSlices* in parallel) and should be neglected (rotation is at constant 400 RPM). Capillary siphon priming is not recorded as it takes place at rest, which does not allow image acquisition.
(MPG)
