# Supplementary material for: Automated Forensic Animal Family Identification by Nested PCR and Melt Curve Analysis on an Off-the-Shelf Thermocycler Augmented with a Centrifugal Microfluidic Disk Segment
Source: PLoS One. 2015 Jul 6;10(7):e0131845. doi: 10.1371/journal.pone.0131845 (PMC4492497; doi:10.1371/journal.pone.0131845)
Supplement: S1 Fig — The strobe RGQ allows acquisition of stationary images of the rotating GeneSlices at arbitrary azimuthal position. It consists of an add-on with a camera, which can be positioned in x- and y-direction. A 45 degree mirror allows observation of the GeneSlices from underneath. (PDF) [file pone.0131845.s001.pdf]

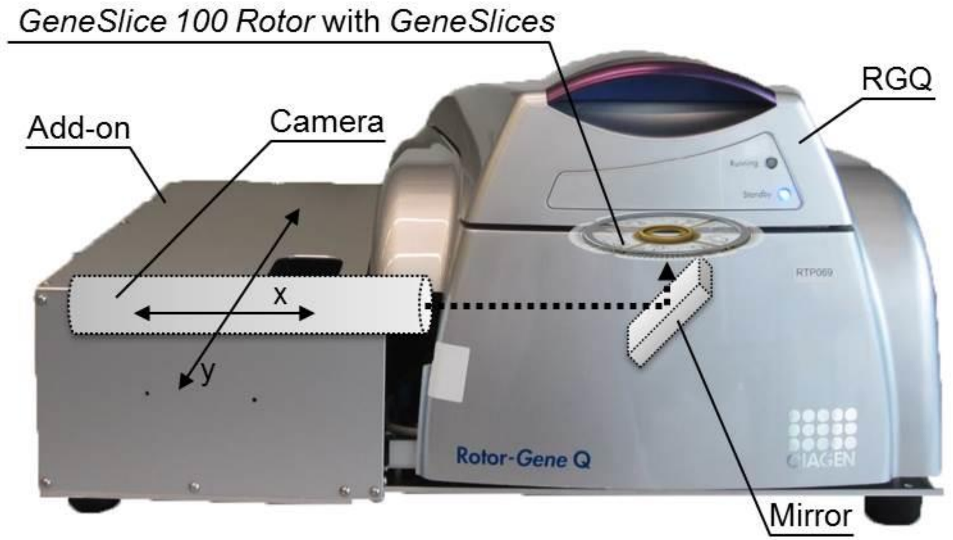

Figure S1: Strobe RGQ. The strobe RGQ allows acquisition of stationary images of the rotating *GeneSlices* at arbitrary azimuthal position. It consists of an add-on with a camera, which can be positioned in x- and y-direction. A 45 degree mirror allows observation of the *GeneSlices* from underneath.
