# Supplementary material for: Automated Forensic Animal Family Identification by Nested PCR and Melt Curve Analysis on an Off-the-Shelf Thermocycler Augmented with a Centrifugal Microfluidic Disk Segment
Source: PLoS One. 2015 Jul 6;10(7):e0131845. doi: 10.1371/journal.pone.0131845 (PMC4492497; doi:10.1371/journal.pone.0131845)
Supplement: S2 Fig — Fluorescence signal increase during pre-amplification indicates that the corresponding main-amplification cavity was filled by premature siphon valving (left). If signals do not increase during aliquoting, it indicates that capillary siphon valving did not take place for the corresponding structure (right). (PDF) [file pone.0131845.s002.pdf]

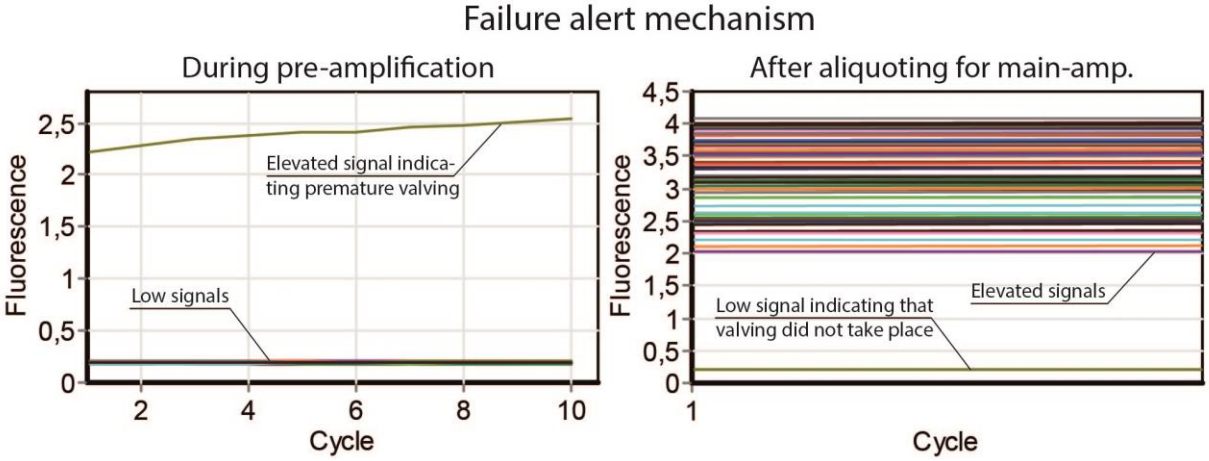

Figure S2: Failure alert mechanism: Fluorescence signal increase during pre-amplification indicates that the corresponding main-amplification cavity was filled by premature siphon valving (left). If signals do not increase during aliquoting, it indicates that capillary siphon valving did not take place for the corresponding structure (right).
