# Supplementary material for: Automated Forensic Animal Family Identification by Nested PCR and Melt Curve Analysis on an Off-the-Shelf Thermocycler Augmented with a Centrifugal Microfluidic Disk Segment
Source: PLoS One. 2015 Jul 6;10(7):e0131845. doi: 10.1371/journal.pone.0131845 (PMC4492497; doi:10.1371/journal.pone.0131845)
Supplement: S3 Fig — If a liquid plug is unintentionally present in the venting channel, an enclosed air volume (light red) is expanded during temperature increase of the hot start for PCR. As a result, the liquid (dark blue) is displaced through the siphon valve (light blue) during pre-amplification. (PDF) [file pone.0131845.s003.pdf]

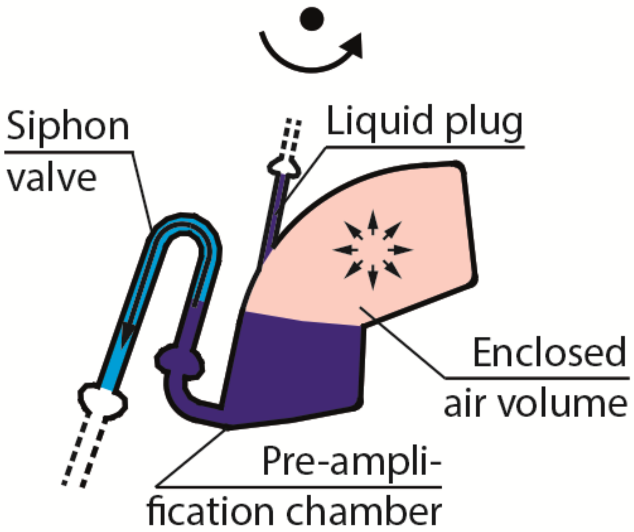

Figure S3: Explanation for premature valving. If a liquid plug is unintentionally present in the venting channel, an enclosed air volume (light red) is expanded during temperature increase of the hot start for PCR. As a result, the liquid (dark blue) is displaced through the siphon valve (light blue) during pre-amplification.
