# Supplementary material for: Automated Forensic Animal Family Identification by Nested PCR and Melt Curve Analysis on an Off-the-Shelf Thermocycler Augmented with a Centrifugal Microfluidic Disk Segment
Source: PLoS One. 2015 Jul 6;10(7):e0131845. doi: 10.1371/journal.pone.0131845 (PMC4492497; doi:10.1371/journal.pone.0131845)
Supplement: S4 Fig — The analysis reveals the occurrence of human DNA and DNA from a species of the family of Canidae. Characterization of the melt peaks identified the DNA origin as fox, which matches an earlier conducted and published examination of the sample with additional species-specific PCR. Controls included in the GeneSlice showed valid signals. (PDF) [file pone.0131845.s004.pdf]

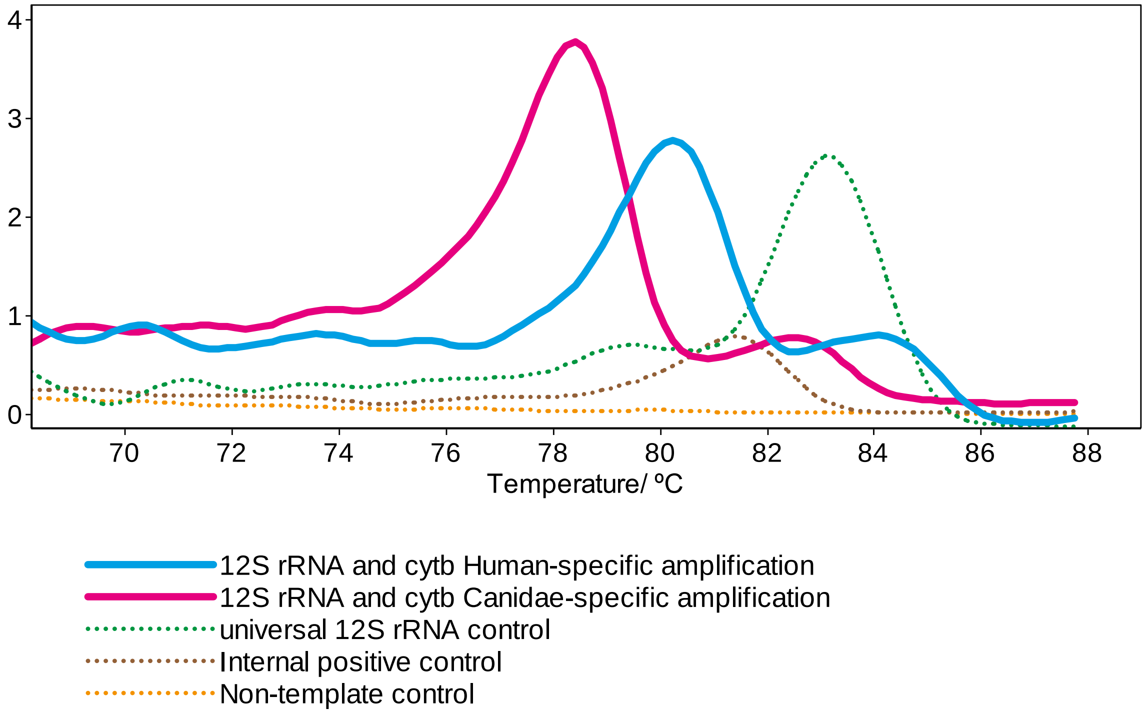

Figure S4: Melt curve analysis after PCR with a *GeneSlice* of DNA recovered from an animal's bite mark on a tent. The analysis reveals the occurrence of human DNA and DNA from a species of the family of Canidae. Characterization of the melt peaks identified the DNA origin as fox, which matches an earlier conducted and published examination of the sample with additional species-specific PCR [1]. Controls included in the *GeneSlice* showed valid signals.

1. Naue J, Lutz-Bonengel S, Pietsch K, Sänger T, Schlauderer N, Schmidt U. Bite through the tent. Int J Legal Med. 2012 May 1;126(3):483–488.
