# Supplementary material for: Automated Forensic Animal Family Identification by Nested PCR and Melt Curve Analysis on an Off-the-Shelf Thermocycler Augmented with a Centrifugal Microfluidic Disk Segment
Source: PLoS One. 2015 Jul 6;10(7):e0131845. doi: 10.1371/journal.pone.0131845 (PMC4492497; doi:10.1371/journal.pone.0131845)
Supplement: S1 Table — Universal 12S rRNA and cytb for pre-amplification were pre-stored within the Sample and NTC pre-amplification chambers. The 12S rRNA and cytb animal-group-specific primers of the twelve groups, as well as the universal 12S rRNA primer pair and internal positive control (IPC) consisting of a primer pair and an 81 bp oligonucleotide were pre-stored in the 14 main-amplification cavities of the sample. The universal 12S rRNA primer pair was additionally used for the main-amplification cavitiy of the NTC. (PDF) [file pone.0131845.s005.pdf]

Table S1: Primer sequences and amount used for pre-storage in the *GeneSlice*. Universal *12S rRNA* and *cytb* for pre-amplification were pre-stored within the Sample and NTC pre-amplification chambers. The *12S rRNA* and *cytb* animal-group-specific primers of the twelve groups, as well as the universal *12S rRNA* primer pair and internal positive control (IPC) consisting of a primer pair and an 81 bp oligonucleotide were pre-stored in the 14 main-amplification cavities of the sample. The universal *12S rRNA* primer pair was additionally used for the main-amplification cavitiy of the NTC.

Cavity	Function	Gene	PCR product (bp)	Primer direction	Primer sequence	Preloaded primer (pmol)
Sample/ NTC	**Pre-amplification**	*12S rRNA*	~200	for	GCCACCGCGGTC *1a	10 (NTC: 2.5)
				rev	TGGGGTATCTAATCCCAG *1a	10 (NTC: 2.5)
		cytb	~ 224	for	CCATGAGGICAAATATCITTITG *1b	40 (NTC: 10)
				rev	GGGTTGTTIGATCCIGTTTC *1b	40 (NTC: 10)
	**Nested PCR *2**					
1	Equidae	*12S rRNA*	172	for	**AAGCGTGATATTGCTCTTTCGTATAG**CTCCGGCGTAAAGCGTGTCAAAGAC	4
				rev	**ACATAGCGACAGATTACAACATTAGTATTG**GTCAGAGGTATTAAAGTCACTTTCGTAG	4
		cytb	128	for	**GTAAAACGACGGCCAGTGA**ATCAGCAATTCCCTACATCGGTAC	4
				rev	**GGAAACAGCTATGACCATG**GCAAAAAATCGGGTAAGGGTG	4
2	Phasianidae	*12S rRNA*	134	for	**AAGCGTGATATTGCTCTTTCGTATAG**GCGTAAAGAGTGGCCACATG	0.5
				rev	**ACATAGCGACAGATTACAACATTAGTATTG**AGGTGGATCTTGGGCTTATGACAGC	0.5
		cytb	141	for	**GTAAAACGACGGCCAGTGA**ATCACAAACCTATTCTCAGCAAT	1
				rev	**GGAAACAGCTATGACCATG**CGAAGAATCGGGTGAGGGTTGGG	1
3	Felidae	*12S rRNA*	168	for	**AAGCGTGATATTGCTCTTTCGTATAG**AACCCAAACTAATAGACCCACGG	3
				rev	**ACATAGCGACAGATTACAACATTAGTATTG**CGTGCTGTATTTTTATGTTAACTGTAGCT	3
		cytb	147	for	**GTAAAACGACGGCCAGTGA**AGAATGAATCTGAGGGGGCTTC	3
				rev	**GGAAACAGCTATGACCATG**GAGGTGTACTGCTGCTAGGGCTGA	3
4	Homo Sapiens	*12S rRNA*	155	for	**AAGCGTGATATTGCTCTTTCGTATAG**AGTGTTTTAGATCACCCCCTCCCC	1
				rev	**ACATAGCGACAGATTACAACATTAGTATTG**ATGTTAAAGCCACTTTCGTAGT	1
		cytb	141	for	**GTAAAACGACGGCCAGTGA**GGCCACAGTAATTACAAACTTACTATC	2.5
				rev	**GGAAACAGCTATGACCATG**TGTGAGGGTGGGACTGTCTACTGAGTA	2.5
5	Sus scrofa	*12S rRNA*	198	for	**AAGCGTGATATTGCTCTTTCGTATAG**TTAACCCAAATTAATAGATCCACGGCGT	2
				rev	**ACATAGCGACAGATTACAACATTAGTATTG**TCGTGTGTCAGGATTATTAGAGTCACT	2
		cytb	135	for	**GTAAAACGACGGCCAGTGA**TACGGTCATCACAAATCTACTATCA	2
				rev	**GGAAACAGCTATGACCATG**GAGGGTTGCTTTGTCGACGGAAAAGCCC	2
6	Cervidae	*12S rRNA*	150	for	**AAGCGTGATATTGCTCTTTCGTATA**GCCCAAGTTAATAGGCACACGGCG	1.5
				rev	**ACATAGCGACAGATTACAACATTAGTATTG**TGGGTCCTAGCTATCGTGTTTCA	1.5
		cytb	98	for	**GTAAAACGACGGCCAGTGA**GAGGAGGCTTTTCAGTAGACAAAGCAACC	2
				rev	**GGAAACAGCTATGACCATG**TAAGTGTACTATAGCGAGTGCTGCGA	2
7	Bovini	*12S rRNA*	137	for	**AAGCGTGATATTGCTCTTTCGTATAG**GCACCATACCAAATAGGGTTAAATTCT	3
				rev	**ACATAGCGACAGATTACAACATTAGTATTG**GGGTCACTTTCGTCATTTATTTTTA	3
		cytb	159	for	**GTAAAACGACGGCCAGTGA**CCCATACATCGGCACAAATTTAGTC	2
				rev	**GGAAACAGCTATGACCATG**GGCAATTGCTATGATGATAAATGGAAGGA	2
8	Canidae	*12S rRNA*	189	for	**AAGCGTGATATTGCTCTTTCGTATAG**CTAATAGGCCTACGGCGTAAAG	1
				rev	**ACATAGCGACAGATTACAACATTAGTATTG**ATCGTGTAGTCAGATTATTATAAAGTCAC	1
					**ACATAGCGACAGATTACAACATTAGTATTG**ATCGTGTAATCAGAAAATTTTAAAGTCAC	1
		cytb	133	for	**GTAAAACGACGGCCAGTGA**ACTAATCTTCTCTCTGCCATCCC	0.5
					**GTAAAACGACGGCCAGTG**AACAAATCTTCTATCTGCTATCCC	0.5
				rev	**GGAAACAGCTATGACCATG**AATCGTGTTAGGGTTGCTTTGTC	0.5
9	Leporidae	*12S rRNA*	159-160	for	**AAGCGTGATATTGCTCTTTCGTATAG**CCGGCGTAAAGCGTGATTAGAATA	1.5
				rev	**ACATAGCGACAGATTACAACATTAGTATTG**AAATCACTTTCGTTGTTTATTTTTGT	1.5
		cytb	117	for	**GTAAAACGACGGCCAGTGA**AACAACCCTAGTTGAATGAATCTGAGGA	1.5
				rev	**GGAAACAGCTATGACCATG**GAAGTGGAAAGCGAAGAATCGG	1.5
10	Mustelidae	*12S rRNA*	132-133	for	**AAGCGTGATATTGCTCTTTCGTATAG**ACGGCGTAAAACGTGTTAAG	1
				rev	**ACATAGCGACAGATTACAACATTAGTATTG**TATTAACAGTAGCTTTTTACGGCCT	2
		cytb	175	for	**GTAAAACGACGGCCAGTGA**GCAACCGTAATTACCAACTTACTGTC	3
					**GTAAAACGACGGCCAGTGA**GCAACCGTAATTACATAACTTATTGTC	3
				rev	**GGAAACAGCTATGACCATG** ATGAATGGTAGGATGAAGTGGAA	2
					**GGAAACAGCTATGACCATG**ATGAATGGCAGGATAAAGTGGAA	2
11	Rodentia (Muridae/Sciuridae)	*12S rRNA*	150-151	for	**AAGCGTGATATTGCTCTTTCGTATAG**CCAAACTAATTATCTTCGGCGTAAA	2
					**AAGCGTGATATTGCTCTTTCGTATAG**CCTAGTTAATAAAGCACGGCGTAA	2
				rev	**ACATAGCGACAGATTACAACATTAGTATTG**GGTCCTAACAATGAATTTTCACATATAAG	3
					**ACATAGCGACAGATTACAACATTAGTATTG**TTATGTTAACTAAGGCTTTTTACAG	3
		cytb	144-146	for	**GTAAAACGACGGCCAGTGA**CGCCACAGTTATTACAAACCTC	0.5
				rev	**GGAAACAGCTATGACCATG**GCGAAGAATCGTGTTAGAGTTGC	1
					**GGAAACAGCTATGACCATG**GAAGAATCGGGTCAAGGTGGC	1
12	Caprinae	*12S rRNA*	185	for	**AAGCGTGATATTGCTCTTTCGTATAG**CGTAAAGCGTGTTAAAGCATCATACT	1
					**AAGCGTGATATTGCTCTTTCGTATAG**CGTAAAACGTGTTAAAGCACTACATC	1
				rev	**ACATAGCGACAGATTACAACATTAGTATTG**TGGGTCTTAGCTATGGTGTATCAG	1
		cytb	152	for	**GTAAAACGACGGCCAGTGA**TATATTGGCACAAACCTAGTCGAA	0.5
				rev	**GGAAACAGCTATGACCATG**GAGGGCTGTGATGATGAATGGG	1
13	**Extraction Control**	*12S rRNA*	~254	for	**AAGCGTGATATTGCTCTTTCGTATAG**GCCACCGCGGTCATACGATT *1	2
				rev	**ACATAGCGACAGATTACAACATTAGTATTG**GGGTATCTAATCCCAGTTTGGGTCTTAGC *1	2
14	**IPC**	Primer *3	119	for	**GTAAAACGACGGCCAGTGA**AAGCGTGATATTGCTCTTTCGTATAG	1
				rev	**GGAAACAGCTATGACCATG**ACATAGCGACAGATTACAACATTAGTATTG	1
		Oligo. *3		81 bp oligo.	AAGCGTGATATTGCTCTTTCGTATAGTTACCATGGCTCAATGCTTACAGAACAATACTAATGTTGTAATCTGTCGCTATGT	100.000 copies

*1:[1], *1a: modified from [1]), *1b: modified from [2] , *2: modified from [3], *3:[4].

Tag on *12S rRNA* primer: IPC forward and reverse primer sequence, respectively.

Tag on *cytb* primer: M13 forward and reverse primer sequence, respectively from the TOPO TA cloning kit (Life Technologies).

1. Tobe SS, Linacre AMT. A technique for the quantification of human and non-human mammalian mitochondrial DNA copy number in forensic and other mixtures. Forensic Sci Int Genet. 2008 Sep;2(4):249–256.
2. Alonso A, Albarran C, Martín P, García P, Capilla J, García O, et al. Usefulness of microchip electrophoresis for the analysis of mitochondrial DNA in forensic and ancient DNA studies. Electrophoresis. 2006 Dec;27(24):5101–5109.
3. Naue J, Lutz-Bonengel S, Sänger T, Schlauderer N, Schmidt U. Modular real-time PCR screening assay for common European animal families. Int J Legal Med. 2014 Jan;128(1):11–18.
4. Swango KL, Timken MD, Chong MD, Buoncristiani MR. A quantitative PCR assay for the assessment of DNA degradation in forensic samples. Forensic Sci Int. 2006 Apr 20;158(1):14–26.
