# Supplementary material for: Automated Forensic Animal Family Identification by Nested PCR and Melt Curve Analysis on an Off-the-Shelf Thermocycler Augmented with a Centrifugal Microfluidic Disk Segment
Source: PLoS One. 2015 Jul 6;10(7):e0131845. doi: 10.1371/journal.pone.0131845 (PMC4492497; doi:10.1371/journal.pone.0131845)
Supplement: S2 Table — Left: Tm of amplification of 1 ng of genomic template DNA; Right: Tm of amplification of the minimal needed DNA amount for successful peak detection (cf. Table 1 for template amount), the coefficient of variation (CV) is given for one representative per group; as these were analyzed in triplicates. (PDF) [file pone.0131845.s006.pdf]

Table S2: Melting temperatures (T_m) of amplimers, as *e.g.* depicted in Figure 3 using different amounts of DNA. Left: T_m of amplification of 1 ng of genomic template DNA; Right: T_m of amplification of the minimal needed DNA amount for successful peak detection (*cf.* Table 1 for template amount), the standard deviation (SD) is given for one representative per group; as these were analyzed in triplicates.

	Melting temperature in °C of			
	1 ng template DNA		20 pg - 200 pg template DNA (species dependent sensitivity level)	
Species	*12S rRNA*	*cytb*	*12S rRNA* ± SD	*cytb* ± SD
Human	80.3	84.4	80.7 ± 0.1	84.3 ± 0.3
Horse	80.3	83.9	80.4 ± 0.4	83.7 ± 0.3
Donkey	79.7	84.0	79.9	84.1
Chicken	82.4	85.3	82.5 ± 0.3	85.7 ± 0.3
Turkey	81.1	85.0	81.0	85.0
Dog	78.8	83.5	79.1 ± 0.3	83.5± 0.0
Fox	78.4	82.6	78.8	82.9
Hare	78.8	82.4	79.1	82.8
Rabbit	78.2	81.9	78.7 ± 0.1	81.9 ± 0.1
Cow	78.8	82.8	78.7 ± 0.1	82.9 ± 0.4
Pig	78.3	83.9	78.8 ± 0.1	84.2 ± 0.1
Cat	79.6	84.0	80.0 ± 0.2	84.2 ± 0.1
Lynx	79.3	85.5	79.7	84.4
Pine marten	78.5	85.3	79.1 ± 0.3	85.5 ± 0.2
Weasel	78.0	83.9	78.8	84.4
Red deer	80.0	82.8	80.0 ± 0.5	83.5 ± 0.4
Roe deer	79.1	82.8	80.1	83.5
Nude mouse	77.3	83.8	77.7 ± 0.1	84.2 ± 0.2
Squirrel	79.0	82.8	79.7	83.4
Sheep	77.6	83.0	78.5	83.7
Goat	79.0	84.3	79.6 ± 0.4	84.4 ± 0.2
