# Supplementary material for: Automated Forensic Animal Family Identification by Nested PCR and Melt Curve Analysis on an Off-the-Shelf Thermocycler Augmented with a Centrifugal Microfluidic Disk Segment
Source: PLoS One. 2015 Jul 6;10(7):e0131845. doi: 10.1371/journal.pone.0131845 (PMC4492497; doi:10.1371/journal.pone.0131845)
Supplement: S3 Table — Comparison between reference and GeneSlice analyses. Tm: Melting temperature. (PDF) [file pone.0131845.s007.pdf]

**Table S3: Summary of results of forensic case samples.** Comparison between reference and *GeneSlice* analyses. $T_m$: Melting temperature.

| Case sample | Reference analysis | | *GeneSlice* analysis | |
|---|---|---|---|---|
| | **Methods *** | **Results** | **Results** | **Interpretation** |
| **Bone sample (spine)** | (1) | **Sheep** DNA | *12s rRNA/ cytb* signal with Caprinae-specific primers; universal *12S rRNA* signal | Member of the sub-family Caprinae; **sheep** due to $T_m$ |
| **"Biltong" sample (meat)** | (1) | **Kangaroo** DNA | no family-specific signal; universal 12S rRNA signal | sample of an animal not covered by the assay |
| **Piece of a tent (fabric) [1]** | (1), (2); species-specific PCR, cloning | Mixture of **fox** and **human** DNA | *12s rRNA/ cytb* signal with Canidae- and human-specific primers; universal *12S rRNA* signal (double peak indicating mixture) | mixed sample: 1. **Human**; 2. Member of the family Canidae; **fox** DNA according to $T_m$ of *cytb* differentiable to dog |
| **Swab from a jacket (skin/blood mixture)** | (1), (2) and subsequent sequencing | No result for universal 12S rRNA/cytb sequencing; Human, cow, pig, chicken after sequencing of human-, Bovini-, pig- and Phasianidae-specific PCR products | *12s rRNA/* low *cytb* signal with human-specific primers; low *12S rRNA* signal with Bovinae-specific primers; universal *12S rRNA* signal | **Human** DNA with a low level of **cow** DNA |

* Methods were chosen according to the task and extended if required by the obtained results

(1) universal *12S rRNA* (404-409bp) and *cytb* (483-488-bp) sequencing [2]; cf. Table S1

(2) animal-group-specific PCR with subsequent melt curve analysis using tubes


1. Naue J, Lutz-Bonengel S, Pietsch K, Sänger T, Schlauderer N, Schmidt U. Bite through the tent. Int J Legal Med. 2012 May 1;126(3):483–8.
2. Naue J, Lutz-Bonengel S, Sänger T, Schlauderer N, Schmidt U. Modular real-time PCR screening assay for common European animal families. Int J Legal Med. 2014 Jan;128(1):11–8.
