# Supplementary material for: Automated Forensic Animal Family Identification by Nested PCR and Melt Curve Analysis on an Off-the-Shelf Thermocycler Augmented with a Centrifugal Microfluidic Disk Segment
Source: PLoS One. 2015 Jul 6;10(7):e0131845. doi: 10.1371/journal.pone.0131845 (PMC4492497; doi:10.1371/journal.pone.0131845)
Supplement: S1 Text — Detailed technical information about the processing steps and materials are presented. (PDF) [file pone.0131845.s008.pdf]

S1 Text: Fabrication of the microfluidic disk segment and pre-storage of reagents. Detailed technical information about the processing steps and materials are presented.

Manufacturing of the computer-aided *GeneSlice* design:

The computer-aided design (CAD) of the *GeneSlice* was CNC micro-milled (Micro MMP 2522, KERN Microtechnik GmbH, Eschenlohe, Germany) four times in a circular pattern into Poly(methyl methacrylate) (PMMA) plates (6 mm thickness, Evonik Industries, Essen, Germany). The resulting negative master was casted with Polydimethylsiloxane (PDMS) (ELASTOSIL RT 607, Wacker Chemie, Munich, Germany) to create a positive master for microthermoforming by soft lithography (µTSL) as replication process [1]: A 188 µm thick cyclo olefin polymer foil (ZEONOR COP ZF 14, Zeon Chemicals, Louisville, KY, USA) was microthermoformed (HEX 01, Jenoptik, Jena, Germany) onto the PDMS master, thereby replicating its structures into the foil. The structured foil was cleaned using 2-propanol and deionized (DI) water and dried.

Application of hydrophilic coating to capillary siphons:

The capillary siphon valve of the sample (and NTC) fluidic path was rendered hydrophilic by pipetting Vistex (111-50, FSI Coating Technologies, Irvine, CA, USA) with 2-propanol 1:40 (1:30) v/v into the region in-between the first two capillary valves, followed by a curing at 120 °C for 60 min [2].

Reagent mixes and pre-storage:

The following reagent mixes for the sample and NTC pre-amplification were prepared and pre-stored into the sample and NTC pre-amplification chamber, respectively: Sample: forward and reverse universal primers (*12S rRNA*, 5 pmol each and *cytb*, 10 pmol each), 1.0 µmol trehalose (Carl Roth, Karlsruhe, Germany), and 10-fold ROX (Jena Bioscience, Jena, Germany); NTC: forward and reverse universal primers (*12S rRNA*, 2 pmol each and *cytb*, 4 pmol each), 0.4 µmol trehalose, 4-fold ROX.

The main-amplification cavities were provided with either animal-group-specific primers, a universal *12S rRNA* or with an 81 bp long oligonucleotide with specific primers as internal positive control as given in S1 Table. Each of the 14 cavities (and NTC cavity) was also supplied with 0.1 µmol trehalose and 2x EvaGreen (Jena Bioscience) for the experiments. Afterward, reagents were air-dried at room temperature overnight in a light-protected and humidity-reduced environment.

1. Focke M, Kosse D, Al-Bamerni D, Lutz S, Müller C, Reinecke H, et al. Microthermoforming of microfluidic substrates by soft lithography (µTSL): optimization using design of experiments. J Micromechanics Microengineering. 2011 Nov 1; 21(11):115002.
2. Focke M, Stumpf F, Roth G, Zengerle R, von Stetten F. Centrifugal microfluidic system for primary amplification and secondary real-time PCR. Lab Chip. 2010 Dec 7; 10(23):3210.
